# Pulmonary Phthisis

**Published:** 1871-08

**Authors:** Austin Flint


### Pulmonary Phthisis.

BY AUSTIN FLINT, M. D.

(The Medical Record, November 15th,)

At a meeting of the New York Academy of Medicine, on Oct. 20th, Dr. Austin Flint, sen., read an instructive paper on "Recent Views in Relation to Pulmonary Tuberculosis," of which the following is an abstract:—

The questions to be thus considered may be divided into those relating, first, to the tuberculous products, and tuberculization; second, to the causation of pulmonary tuberculosis; and third, to the treatment.

The speaker stated clearly the prominent late views relating to the morbid products which for more than half a century have been called tuberculous, and to tuberculization—these views were from a histological stand-point, and spoke as follows:—

To discuss their merits might seem presumptuous in one who is not a practical histologist. But the great majority of those who are expected either to adopt or reject views founded on histological data are not, and cannot be, workers with the microscope. They cannot be expected to confirm or correct these data by their own observations. They must be content to compare the testimony of different observers, and judge for themselves according to their confidence in the observers. They may be competent, however, to exercise their judgment as to whether the data are sufficient to warrant the views which are thereon based. They may even be better qualified to do this than those who draw deductions from original observations, for experience has shown abundantly that microscopical observations are peculiarly exposed to error from the bias of speculation or preconceived convictions.

With respect to purely histological points, there seems to be, for the most part, agreement among practical histologists. Different microscopical observers do not differ as to the appearances under the microscope. But there is not, as has been seen, the same agreement concerning the conclusions: there is much discrepancy in the interpretation of the appearances. Virchow and the disciples of the "cellular pathology" interpret certain anatomical elements to mean proliferation of normal cells. Robin, Bennett, and others, explain their production by a spontaneous generation. The cheesy metamorphosis, tyrosis, or granulo-fatty degeneration, of which so much has been said of late years, is perhaps inferred rather than proved; it is a mooted point as to the inflammatory or non-inflammatory character of the tuberculous granulations now recognized by many as the only true tubercles. Admitting that the so-called tuberculous infiltration is an inflammatory product, it is not certain that the primary process is an inflammation; at all events, there is room here for doubt and discussion; the relationship between the two products just named is a topic for directly opposite opinions. It is plain that the revelations of the microscope, valuable as they are, have not, as yet, been sufficient to settle these and other pathological questions. Much stress is laid by recent observers on the essential difference between these two products, yet one of the most distinguished of those engaged in the study of tubercle, namely, Villemin, declares that he "thinks" (to quote his own words) "there is ground for returning to the ideas of the great Laennec and the judicious Andral in behalf of the unity of tuberculosis."

It is deemed a highly important object, as a result of late histological researches, to call the affection heretofore known as infiltrated tubercle, a pneumonia. Let it be granted that there is a strict pathological propriety in this, the practical advantage which is gained is not very apparent. The long established points in morbid anatomy which distinguish this affection from other forms of pneumonia remain unchanged. Here is a product which is absorbed with great difficulty, if at all, whereas the intra-vesicular product in ordinary pneumonia is generally, and often rapidly, removed by absorption. The former leads to abscesses, and to cavities which very rarely cicatrize; the latter seldom ends in abscesses, and when they occur the cavities resulting from them tend to cicatrization. The tuberculous pneumonia is a bilateral affection, the instances in which it is confined to one lung being rare exceptions to the rule; the reverse of ordinary pneumonia. On the other hand, contrast the tuberculous pneumonia with the chronic form in which the morbid product is interstitial, and these points of difference are not less striking. If it be resolved to call the affection a pneumonia, some qualifying term, denoting its distinctness of character, is certainly important. The word *cheesy*, or the more refined synonym *caseous*, is objectionable on the ground that for it to have any precision, the age and the kind of cheese to which it is analogous should be stated. The name "chronic catarrhal pneumonia" must derive its significance chiefly from the supposition that the pneumonia supervenes upon a bronchitis, which is a supposition, to say the least of it. Tuberculous pneumonia seems the most appropriate term. The term is objected to on the ground that the affection is not always, although often, associated with true tubercles, that is, the gray granulations. To call a pneumonia without tubercles, tuberculous, would, of course, be a misnomer. The objection is a valid one only in view of the hypothesis that tubercles or granulations are consecutive to, and dependent upon, the pneumonia; the latter, accordingly, existing for a time without, and in some cases not followed by the former. Niemeyer carries this hypothesis so far as to say that "the greatest danger for the majority of pneumonia's is, *they are apt to become tuberculous*" —a curious statement when it is considered that, assuming the correctness of the hypothesis, to determine whether the pneumonia be or be not associated with tubercles, is confessedly rarely within the grasp of diagnosis. As a concluding remark, in connection with this division of the subject, the distinctions and names which have lately been introduced can hardly be said to simplify our knowledge of tuberculous disease. I will content myself, in illustration of this remark, with citing the nosological divisions in the late treatise on Pulmonary Phthisis by Hèrard and Cornil, a work of labor and ability, and containing mucn valuable information.

These authors treat of the following forms of disease as entering into the study of pulmonary phthisis:—

1st. Lobar tuberculous pneumonia; 2d. Lobular tuberculous pneumonia; 3d. Generalized granular phthisis; 4th. Generalized granular phthisis, with pneumonia; 5th. Partial granular phthisis; 6th, Acute or galloping phthisis; 7th. Generalized caseous lobar pneumonia. Into these seven varieties are resolved the chronic and acute phthisis of other authors.

*The Causation of Pulmonary Tuberculosis.*—The discovery by Villemin of the inoculability of tubercle stands out in strong relief among the conflicting and perplexing views which have been noticed. The inoculability of tubercle is a positive indubitable fact, which not unlikely will constitute a highly important epoch in the history of tuberculous disease. Connected with this discovery are certain questions not as yet settled. The specific character of tuberculosis is one of these. In other words, does the matter inoculated, when the disease is thus communicated, contain a special virus? Villemin claims that his experiments prove the affirmative to this question, and establish for tuberculous disease a specific character such as belongs to variola, scarlatina, syphilis, and glanders. His work on tuberculous phthisis is a masterly effort to show this by collateral evidence, as well as by the experiments establishing the inoculability of the disease. On the other hand, Lebert, Wryss, Waldenburg, in Germany; Colin, in France, and Andrew Clarke, Simon, Sanderson, and Wilson Fox, in England, have made experiments which appear to show that inoculation with other than tuberculous products (using this term in its widest sense) is sometimes followed by the development of tubercles in the lungs and different organs. Villemin claims that in these experiments either the products furnishing the matter for inoculation contained the tuberculous virus, or the products following the inoculation were not true tubercles. The settlement of this point by continued researches is evidently of much importance in its bearing on the question as to the specific character of the disease.

From the fact of inoculability springs another question, which needs only be stated for its practical importance to be sufficiently obvious. Inoculation proves contagiousness in the strict and proper adaptation of the latter term; but is not the disease communicable by infection? This is the question now referred to. May not material emanations from tuberculous patients be received into the air-passages, and the disease in this way produced? Contagion does not prove infection—a fact of which syphilis in an exemplification--but taking into view morbid emanations which must be contained in the expired breath of patients affected with tuberculous disease, the possibility, if not probability, of its being sometimes thus communicated becomes an important point for renewed clinical study. And, pending the accumulation and investigation of facts, it is certainly a wise precaution to enjoin upon healthy persons, and those already tuberculous, to avoid, as far as practicable, an atmosphere which, from defective ventilation or the congregation of phthisical patients, or both, must abound in the suspected emanations.

From the time of Laennec a tuberculous diathesis had been supposed to be largely involved in the causation of pulmonary tuberculosis; but, according the views of some distinguished writers of the present day, this diathetic agency has been greatly overestimated. Villemin repudiates altogether the existence of a diathesis which is independent of the introduction within the system of a special virus. Others, for example Niemeyer, while they do not ignore a constitutional predisposition, think that its causative influence is vastly less than has been supposed, and that a proportionately greater agency is exerted by local exciting causes. The advocates of this view consider that an active, special diathesis does not exist, but only a certain amount of vulnerability (to use a word of recent application) arising from an enfeebled power of resistance to the local causes of disease. This view, in fact, almost does away with a diathetic influence, since the term diathesis denotes a predisposition to a particular form of disease. It has been hitherto believed that tuberculous disease is developed chiefly, and sometimes even exclusively, as a result of a special, determining, constitutional, morbid condition. This is the idea attached to the term tuberculous diathesis. Local exciting causes have been regarded as of secondary importance, being never, in themselves, sufficient to produce the disease, and not being included in the causation. Here are questions of great practical importance. The teachings of Niemeyer are in strong contrast with the views which have been, and are still, generally held by physicians; and it is needless to call attention to their direct and important bearing on treatment.

These views are to be sustained, or otherwise, not by arguments, but by evidence derived from clinical experience. Is it true that tuberculous infiltration, or pneumonia, as determined by physical signs, is preceded generally or frequently by bronchitis? The answer to this question in the negative purports to be based on clinical facts. So far as one can judge from publications, the affimative, if not merely a speculative or rational conclusion, is supported, as yet, only by the impressions formed on a limited experience.

The latter answer is a revival of an old belief which has never been eliminated from the popular mind. If the question is to be settled anew, this can only be done fairly by a fresh appeal to clinical observation. It is practicable to settle the question in that way. The existence or non-existence of bronchitis, or a bronchial catarrh, antecedently to the presence of signs denoting tuberculous solidification of lung, is a point which, if the opportunity be offered, a competent clinical observer can determine in individual cases. Until the question is settled, let us not accept the new view because it is affirmed, or because it is thought to harmonize with other views.

The remarks just made are alike applicable to the question whether hæmoptysis stands in a causative relation to tuberculous disease. The affirmation of such a relation, no matter by whom made, and the reasonableness of it, or its consistency with other views, are of no moment except in so far as they are sustained by clinical evidence. The question is to be settled by determining whether hæmoptysis, occurring when tuberculous disease does not exist, is followed by this disease in a proportion of cases sufficiently large to show a relation of causation. Clinical observation is competent to determine this point, and, until this is done, it is the part of prudence and philosophy to hold the mind in abeyance with regard to the adoption of this, as of the other of the views just stated.

*The Treatment of Pulmonary Tuberculosis.*—Within the recollection of the older physicians of the present day, the treatment of pulmonary tuberculosis has undergone an entire change. The measures in vogue thirty or forty years ago were those which have been known as antiphlogistic, namely, venesection, aed local blood-letting, mercurialization, and active counter-irritation. With these were conjoined a restricted diet and confinement within doors.

Impartial observers who are able to compare the success of treatment then and now, must bear testimony to a great improvement. The proportion of recoveries is certainly much greater. When recovery does not take place, the disease is much oftener non-progressive, and, when not stationary, its progress is more slow. Death is not so often preceded by a long period of confinement to the bed and suffering from extreme debility and bed-sores.

In these respects the contrast is very striking, and it is fair to attribute it to the change in treatment. Of late years the so-called antiphlogistic measures have fallen into disuse; and, in place of these, tonic remedies and alcoholic stimulants have been much employed, together with cod-liver oil, a generous diet, and life out of doors.

It is not easy to determine how far the improvement in the success of treatment is due to the abandonment of measures formerly employed, and how far to those which have been substituted, but that the former were injurious can hardly be doubted. Of the measures now in vogue, there may be differences of opinion with reference to the relative importance of those which are, in the strict sense of the term, therapeutical, and those which come under the head of diet and regimen ; but I think few are to be found who do not regard the latter as vastly more important than the former. It is by no means uncommon for cases to be treated exclusively by hygienic measures.

Now, it is proposed to return to some of the measures which had become most obsolete in practice.

In the recent treatises by Hèrard and Cornil, and by Niemeyer, bloodletting is advocated as a measure important in certain cases of the common form of pulmonary tuberculosis, namely, that known as infiltrated tubercle. In the first named of these two treatises, even venesection is recommended, and in the second, local bleeding by cups or leeches. Hèrard and Cornil advocate the use of the tartrate of antimony. They advise confinement within doors during the winter season. Niemeyer goes further, and advises confinement to the bed whenever febrile phenomena are present. These measures of treatment are based on the pathological view which considers infiltrated tubercle as a form of pneumonia; they are employed in other words, as antiphlogistic measures. Hèrard and Cornil state that the therapeutical indications are nearly the same as in cases of frank pneumonia; and Niemeyer states as a reason for giving to the affection the name "chronic catarrhal pneumonia," that thereby "its prophylaxis and therapeusis are promoted."

This new view of the treatment of pulmonary tuberculosis seems to me to illustrate the tendency to engraft therapeutics on pathology. The disease, as it is generally presented in practice, is a pneumonia, and it therefore claims the treatment which is supposed to be appropriate to pneumonic inflammation.

But this is a *non sequitur*, according to the general truth stated in entering on this division of the subject. It does not follow that these measures are indicated, granting that the affection is a form of pneumonia. The only true basis of therapeutics here, as elsewhere, is in clinical experience. Has clinical observation been correct in leading to the conclusion that these measures of treatment are injudicious, at a time when tuberculization was generally thought to be a non-inflammatory process? then, assuredly, this conclusion is not upset by proving that the process is an inflammation. The lessons of experience are not to be subverted by changing the name tuberculous infiltration to catarrhal or tuberculous pneumonia.

It is not, then, an unreasonable scepticism, or conservatism, to protest against the adoption of this new view of treatment on mere pathological grounds. We are bound, as it seems to me, not to adopt it until it is substantiated by a collection of facts obtained by experimental observation. We are bound to adopt it whenever it is so substantiated. They who are fairly convinced of the propriety of these measures, as the result of speculation or reasoning will not be likely to hesitate in employing them; but there are many who cannot at present conscientiously submit them to the test of experimentation.

The pathological opinion stated under the head of causation (and which I believe may be proved to be erroneous), that pulmonary consumption is a sequel of bronchitis, has an important bearing on the treatment. If this opinion be correct, it is a rational inference that it is a special object of treatment to prevent the recurrence of attacks of bronchitis, and that therapeutical measures are to be specially directed to bronchial inflammation. Of those who have had a large experience in the management of the disease in this country, not a few attach most importance to out-of-door life, and enjoin upon patients not to be deterred from carrying out this hygienic measure by apprehensions of "taking cold." It is common to assure patients that the existence of pulmonary tubercles does not render them more liable to attacks of bronchitis, and, if attacks occur, the tuberculous affection is not thereby increased. These statements, which date from the researches of Laennec and Louis, are not in accordance with popular belief, a fact which Niemeyer thinks is very fortunate, considering, as he does, the latter to be the more correct view. This view, there is reason to believe, is based on the supposed causative connection between bronchitis and consumption, rather than on clinical experience.—*Half-Yearly Abstract.*

